# Enhanced anti-ischemic stroke of ZL006 by T7-conjugated PEGylated liposomes drug delivery system

**DOI:** 10.1038/srep12651

**Published:** 2015-07-29

**Authors:** Zhongyuan Wang, Yue Zhao, Yan Jiang, Wei Lv, Lin Wu, Baoyan Wang, Lingyan Lv, Qunwei Xu, Hongliang Xin

**Affiliations:** 1 Department of Pharmaceutics, School of Pharmacy, Nanjing Medical University, Nanjing 211166, China

## Abstract

The treatment for ischemic stroke is one of the most challenging problems and the therapeutic effect remains unsatisfied due to the poor permeation of drugs across the blood brain barrier (BBB). In this study, HAIYPRH (T7), a peptide that targeted to transferrin receptor (TfR) can mediate the transport of nanocarriers across the BBB, was conjugated to liposomes for ischemic stroke targeting treatment of a novel neuroprotectant (ZL006). T7-conjugated PEGylated liposomes (T7-P-LPs) loaded with ZL006 (T7-P-LPs/ZL006) were showed satisfactory vesicle size and size distribution. Furthermore, the cellular uptake results showed that T7 modification increased liposomes uptake by the brain capillary endothelial cells (BCECs) and little cytotoxicity of liposomes with or without ZL006 was observed. The *in vivo* biodistribution and near-infrared fluorescence imaging evidenced that T7 modification rendered liposomes significantly enhanced the transport of liposomes across the BBB. The pharmacodynamic study suggested that, T7-P-LPs/ZL006 exhibited reduced infarct volume and ameliorated neurological deficit compared with unmodified liposomes or free ZL006. T7-P-LPs/ZL006 could be targeted to brain and displayed remarkable neuroprotective effects. They could be used as a potential targeted drug delivery system of ischemic stroke treatment.

The impact of stroke, particularly ischemic stroke, can be devastating, and unlike other disabling neurological diseases, stroke has a high incidence, prevalence and rate of subsequent disability. The sudden onset leaves the individual and the family ill-prepared to deal with their residual impairments of physical, psychological, and social functions^1^,^2^,^3^. Obviously there is an urgent need for an effective treatment for ischemic stroke.

Because of poor tolerance for humans, many drugs have been failed to gain approval for clinical use to treat ischemic stroke^4^. ZL006, 5-(3, 5-dichloro-2-hydroxybenzylamino)-2-hydroxybenzoic acid, was a special uncoupling agent of ischemia-induced reactions screened by our center^5^. It could selectively block the coupling of neuronal nitric oxide synthase and the scaffold protein postsynaptic density 95 kDa, and had potent neuroprotective activity. Moreover, ZL006 has no effect on aggressive behavior and spatial memory. Even so, the available therapeutics has less than optimal usefulness for ischemic stroke, mainly owing to the low permeability across the blood brain barrier (BBB). Although there is a compromised endothelial barrier which facilitates molecular transport under some disease conditions such as Alzheimer’s disease and ischemic stroke, BBB is still present in the infiltrating margin of brain diseases^6^. Consequently, there is an increasing need for novel method to overcome the BBB for the central nervous system diseases treatment.

To solve the poor penetration across BBB, many strategies have been developed these years. Receptor-mediated endocytosis is one of the most important mechanisms of brain targeting drug delivery system. As known, transferrin receptor (TfR) can mediate the transcytosis of transferrin-bound iron. It is over-expressed on the brain capillaries endothelial cells, which makes TfR an attractive target. A special ligand peptide of TfR, HAIYPRH (T7), can specially bind to TfR and mediate the transport of nanocarriers across the BBB^7^,^8^. Recently, T7-modified nanoparticles were reported with good targeting ability to brain tumors^9^,^10^. Moreover, T7-conjugated polyamidoamine dendrimer was used for nanoscale brain-targeted magnetic resonance imaging^11^. Through the T7 peptide modification, nanocarriers could effectively achieve the active targeting to brain site. However, there is no previous study about T7-modified liposomes (T7-P-LPs) utilization on targeted therapy of ischemic stroke through TfR-mediated endocytosis.

With these considerations, we developed T7 peptide modified ZL006 loading liposome (T7-P-LPs/ZL006) to improve the drug delivery to brain and enhance the therapeutic effect against ischemic stroke for ZL006 in this study. Firstly, T7 was conjugated to liposomes, and the morphology, particle size, encapsulation efficiency and loading capacity were investigated. Secondly, the *in vitro* and *in vivo* targeting efficacy of T7-P-LPs were studied *via* cellular and animals model. Finally, the *in vivo* anti-ischemic stroke efficacy of T7-P-LPs/ZL006 was evaluated through rats middle cerebral artery occlusion (MCAO) model.

## Results

### Synthesis of T7-PEG-DSPE

**Figure 1:**
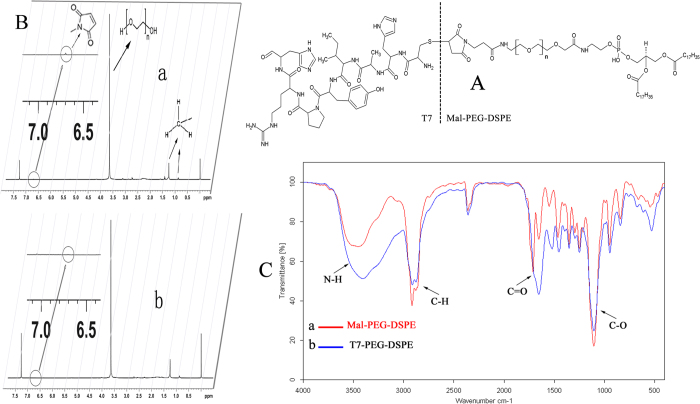
The structure of T7-PEG-DSPE (**A**); ^1^H NMR spectra (**B**) and FTIR spectra (**C**) of Mal-PEG-DSPE (**a**) and T7-PEG-DSPE (**b**).

The structure of T7-PEG-DSPE is shown in Fig. 1A. As the ^1^H NMR spectra of Mal-PEG-DSPE and T7-PEG-DSPE shown in Fig. 1B, the solvent peak of CDCl_3_ was present at 7.26 ppm. The characteristic peak of methylene protons of DSPE, PEG peaks and maleimide group of Mal-PEG-DSPE polymer was at 1.2 ppm, 3.7 ppm and 6.7 ppm, respectively. However, the maleimide peak disappeared in the spectrum of T7-PEG-DSPE, whereas the DSPE and PEG segment still respectively presented sharp peaks at 1.2 and 3.7 ppm, suggesting that T7 was already conjugated with Mal-PEG-DSPE.

The FTIR spectra of Mal-PEG-DSPE and T7-PEG-DSPE are shown in Fig. 1C. The spectrum of Mal-PEG-DSPE showed a weak C=O stretch band at 1686.21 cm^−1^, and a broad N-H stretch band at 3600–3200 cm^−1^, centered at 3403.63 cm^−1^. The two stretch bands are characteristic absorption of the secondary amide groups in the structure of Mal-PEG-DSPE. In the spectrum of T7-PEG-DSPE, the intensity of the two bands remarkably increased, which could be attributed to the seven secondary amide groups in the structure of T7. These results demonstrated the successful synthesis of T7-PEG-DSPE.

### Characterization of liposomes

**Figure 2:**
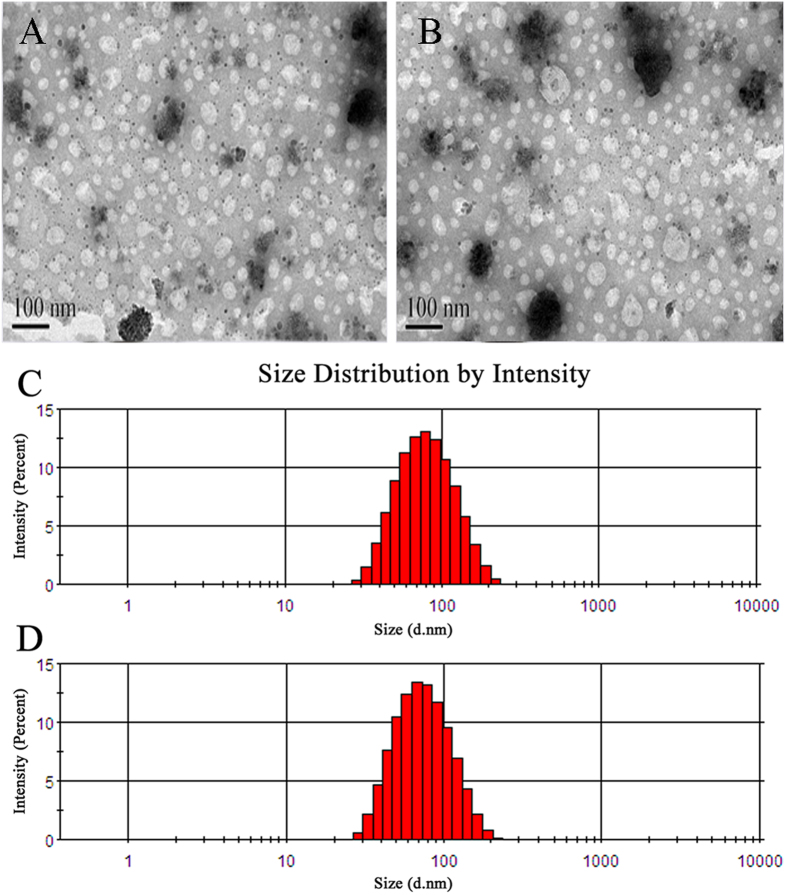
TEM image of P-LPs/ZL006 (**A**) and T7-P-LPs/ZL006 (**B**); particle size and size distribution of P-LPs/ZL006 (**C**) and T7-P-LPs/ZL006 (**D**).

The characterizations of liposomes loaded with ZL006 are shown in Table 1. The liposomes with or without T7 modification showed similar vesicle sizes, polydispersity indexes (PDI), loading capacity (LC) and encapsulation efficiency (EE), indicating that the incorporation of T7-PEG-DSPE into liposomes had no influence on the physical properties of liposomes. The LC of P-LPs/ZL006 and T7-P-LPs/ZL006 were 85.37 ± 2.06% and 87.89 ± 3.58% along with 9.40 ± 0.45% and 9.76 ± 0.55% EE, respectively. In addition, TEM photographs show that P-LPs/ZL006 and T7-P-LPs/ZL006 were generally spherical and of regular size (Fig. 2A,B), consistent with the size distribution (Fig. 2C,D). The characterizations of liposomes exhibited small sizes, evenly distribution, good PDI, regular spherical and high LC, suggesting that such liposomes provide a promising basis in the follow-up experiment.

### Cellular uptake of liposomes by BCECs

**Figure 3:**
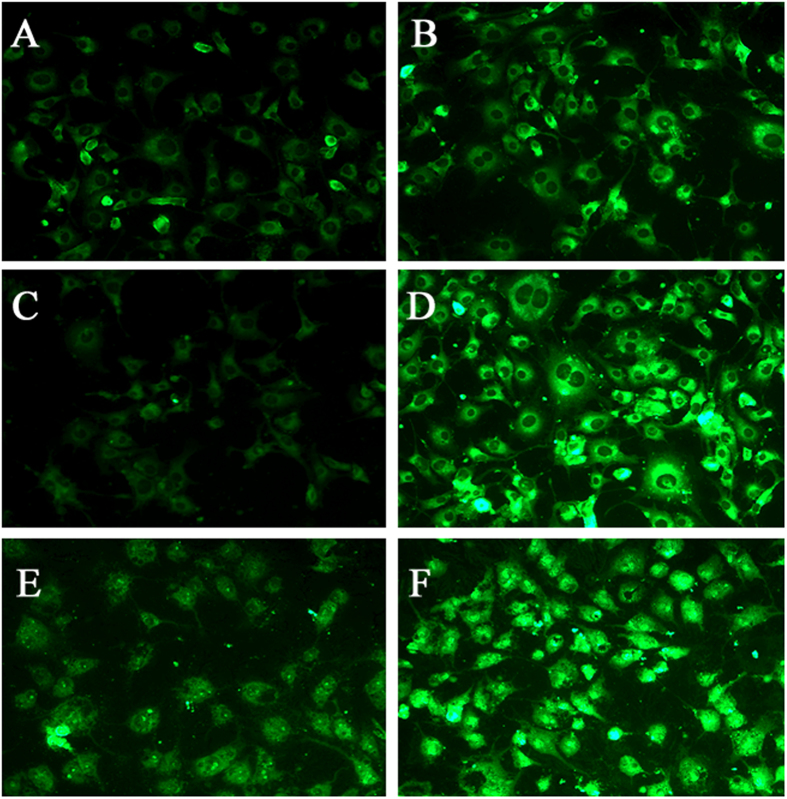
BCECs uptake of coumarin-6-labeled P-LPs (**A,C,E**) and T7-P-LPs (**B,D,F**) at the concentration of 5 ng/mL (**A,B**), 10 ng/mL (**C,D**) and 20 ng/mL (**E,F**) was examined by fluorescent microscopy. Green: coumarin-6 labeled liposomes. P-LPs: coumarin-6-labeled P-LPs; T7-P-LPs: coumarin-6-labeled T7-P-LPs. Original magnification: ×20.

The cellular uptake characteristic of liposomes was investigated qualitatively by fluorescent microscopy. The results shows that BCECs treated with either coumarin-6-labeled P-LPs or T7-P-LPs exhibited fluorescent intensity corresponding to incubation concentration (Fig. 3). The fluorescent intensity of coumarin-6-labeled T7-P-LPs exhibited a concentration-dependent mode and obviously higher than that of P-LPs when the coumarin-6 concentration ranged from 5 ng/mL to 20 ng/mL for 1 h incubation.

**Figure 4:**
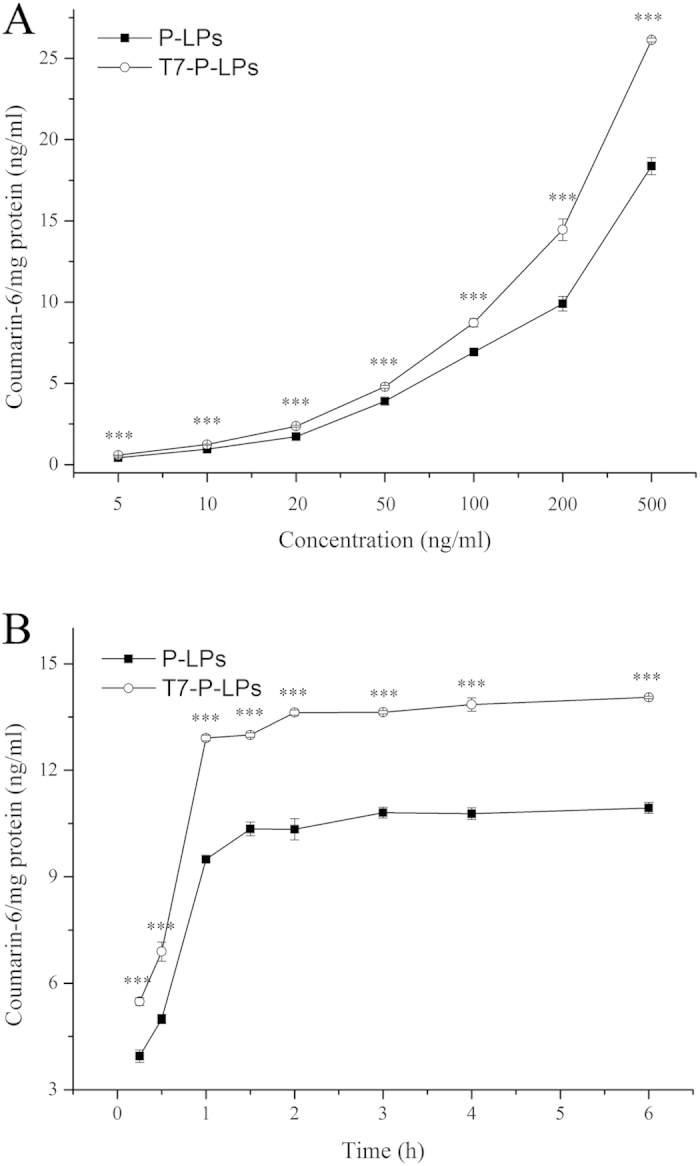
Cellular uptake of coumarin-6-labeled P-LPs and T7-P-LPs after incubation for 1 h at the concentrations ranged from 5 ng/mL to 500 ng/mL in BCECs (**A**). Cellular uptake of coumarin-6-labeled P-LPs and T7-P-LPs at different incubation time ranged from 0.25 to 6 h in BCECs at the concentration of 100 μg/mL (**B**). ***p < 0.001 compared with P-LPs.

Quantitatively, with the T7 peptide modification, the intracellular coumarin-6 intensity of T7-P-LPs was significantly higher than that of unmodified P-LPs at the concentrations ranged from 5 ng/mL to 500 ng/mL, and at the incubation time ranged from 0.25 to 6 h (Fig. 4). The quantitative analysis of cellular uptake by BCECs was also in a concentration-dependent mode within 1 h (Fig. 4A). The cellular uptake of coumarin-6-labeled T7-P-LPs was 1.34, 1.37, 1.46 and 1.42 folds higher when compared with that of P-LPs at the concentration of 5, 20, 200 and 500 ng/mL, respectively. In addition, the time-related experiment exhibited that the fluorescence intensity of coumarin-6-labeled T7-P-LPs on BCECs was significantly enhanced when compared with that of P-LPs at all experiment time points (Fig. 4B). These results indicated that T7 played an active targeting role in T7-P-LPs uptake by BCECs via transferrin receptor mediated endocytosis.

### Quantitative analysis of ZL006 loaded liposomes uptake by BCECs

**Figure 5:**
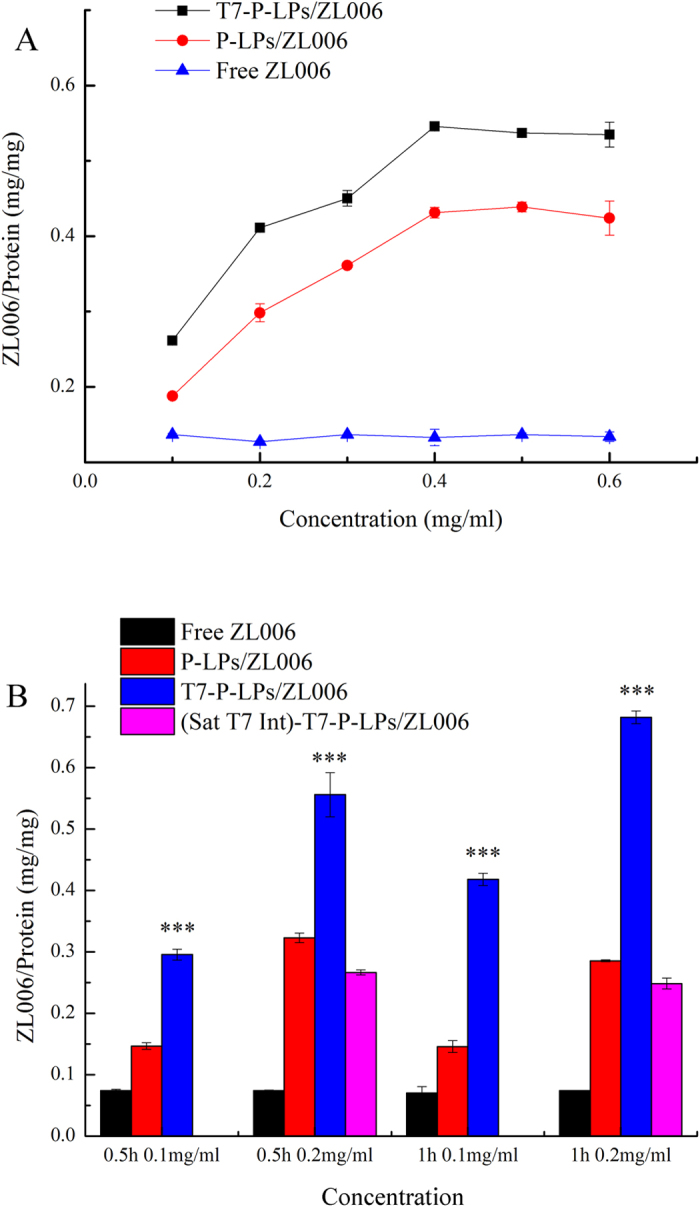
Cellular uptake of ZL006 loaded P-LPs and T7-P-LPs after incubation for 0.5 h at the concentrations ranged from 100 μg/mL to 600 μg/mL in BCECs (**A**). Cellular uptake of T7-P-LPs/ZL006, P-LPs/ZL006 and free ZL006 after incubation for 0.5 h and 1 h with the concentration of 200 μg/mL. Saturated T7 intervened group (Sat-T7-Int)-T7-P-LPs/ZL006 was added at the concentration of 200 μg/mL for 0.5 h and 1 h. ***p < 0.001 compared with P-LPs/ZL006.

We evaluated the uptake of ZL006 loaded T7-P-LPs (T7-P-LPs/ZL006), P-LPs (P-LPS/ZL006) and free ZL006 in different concentration by BCECs depended on the incubation time within 0.5 h (Fig. 5A). It indicated that the uptake of both liposomes were concentration-dependent. The uptake increased with increasing concentration of liposomes, and the uptake of T7-P-LPs/ZL006 reached 1.3 times than that of P-LPS/ZL006 at 400 μg/mL and the uptake of liposomes reached saturation at that concentration. However, with free ZL006, it present a negatively concentration dependent phenomenon at the concentrations ranged from 100 μg/mL to 600 μg/mL. At different time point, the uptake of liposomes was both much higher than that of free ZL006, and the intake of T7-P-LPs/ZL006 was higher than that of P-LPs/ZL006, obviously.

The enhanced uptake of T7-P-LPs/ZL006 than P-LPs/ZL006 by BCECs might be caused by transferrin receptor mediated endocytosis. To confirm this, we added excess free peptide T7 into each well for 30 min incubation. Then T7-P-LPsZL006, P-LPs/ZL006 and free ZL006 were added and incubated for 30 min after removing free peptide T7. From (Fig. 5B), the uptake of T7-P-LPs/ZL006 was significantly higher than T7-P-LPs/ZL006 with free peptide T7 intervened, about 2.08 and 2.74 times in 30 min and 1 h, respectively. And the latter group showed the similar level with P-LPs/ZL006. Besides, there weren’t obviously differences of uptake between P-LPs/ZL006 and free ZL006 with T7 saturated P-LPs/ZL006 and T7 saturated free ZL006, respectively. Meanwhile, the uptake of T7-P-LPs/ZL006 reached 1.72 and 2.38 times higher than that of P-LPs/ZL006 with the concentration of 200 μg/mL at 30 min and 1 h, respectively. In receptor competitive inhibition assay, pre-incubation with free T7 peptides obviously inhibited the cellular uptake of T7-P-LPs, which suggested the endocytosis of T7-P-LPs by BCECs was mediated by transferrin receptor.

### Cytotoxicity of liposomes with or without ZL006

**Figure 6:**
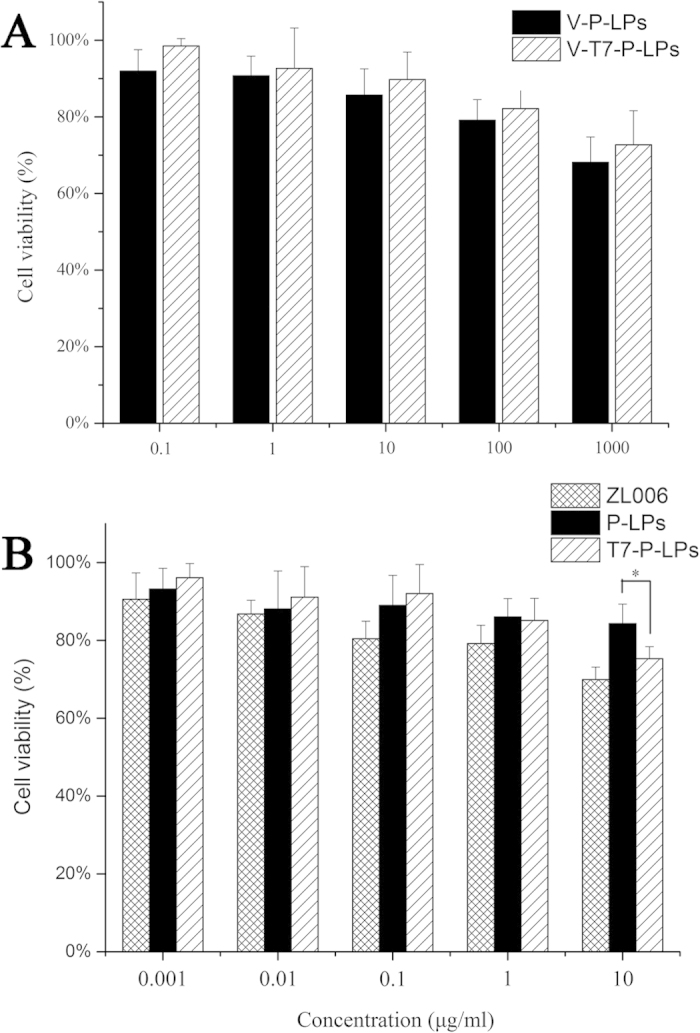
Cell viability of BCECs after treated with blank vehicles (**A**); ZL006, P-LPs/ZL006 and T7-P-LPs/ZL006 (**B**) for 72 h at 37 °C. V-P-LPs: vehicles-P-LPs; V-T7-P-LPs: vehicles-T7-P-LPs; P-LPs: P-LPs/ZL006; T7-P-LPs: T7-P-LPs/ZL006.

As shown in Fig. 6, the *in vitro* toxicity of liposomes with or without ZL006 was evaluated on BCECs by MTT assay. The result displayed that two blank liposomes almost had no toxicity to the BCECs (Fig. 6A). However, the cytotoxicity was increased without significance at high liposomes concentration of 1000 μg/mL. Furthermore, ZL006, as a neuroprotective agent, presented little cytotoxicity, and a growth inhibition of BCECs was not found in all groups at low concentration of 0.001, 0.01, 0.1, 1 and10 μg/mL. Worth noting that the cytotoxicity of T7-P-LPs/ZL006 was significantly enhanced (p < 0.05) at the concentration of 10 μg/mL, indicating that more T7-P-LPs/ZL006 were taken in BCECs and the more drugs may cause more cytotoxicity (Fig. 6B).

### *In vivo* penetration across BBB of T7-P-LPs

**Figure 7:**
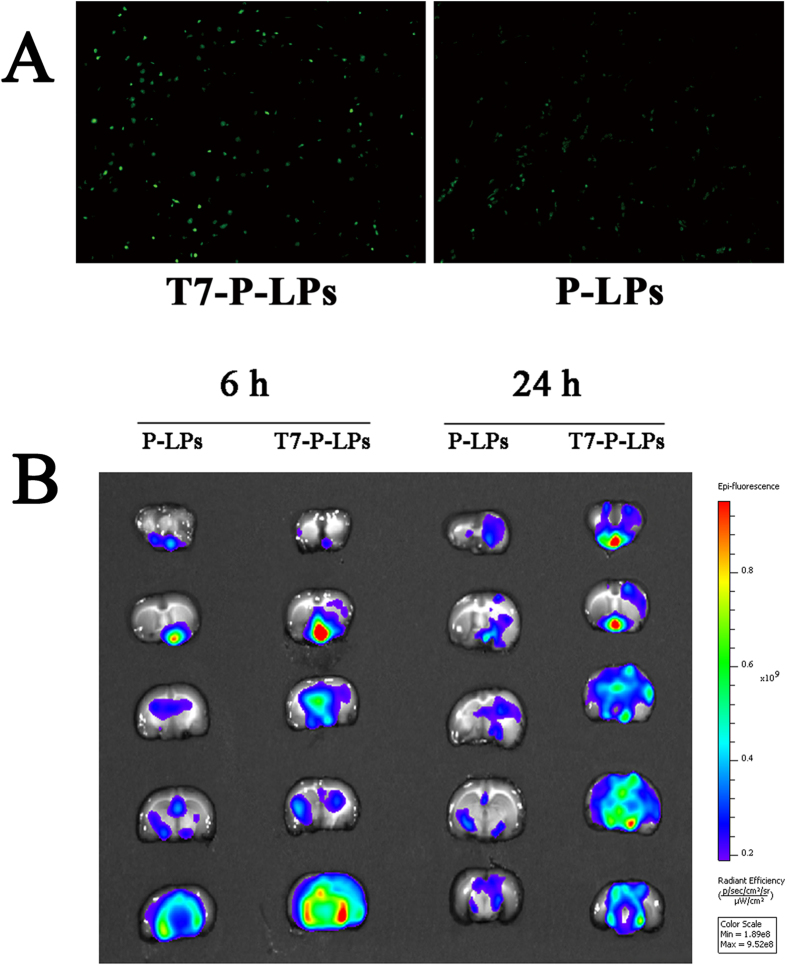
The *in vivo* BBB penetration ability of coumarin-6-labeled T7-P-LPs and P-LPs (**A**). Green: coumarin-6 labeled liposomes. Original magnification: × 10. *Ex vivo* fluorescent image of DiR-labeled P-LPs and T7-P-LPs in the ischemic brain at 6 and 24 h (**B**). Data are represented with mean ± SD (n = 2). P-LPs: DiR-labeled P-LPs; T7-P-LPs: DiR-labeled T7-P-LPs.

The *in vivo* brain targeting capability of coumarin-6-labeled liposomes was studied qualitatively by fluorescence microscopic observation of coronal sections of the rat brain. These results showed that green particles of coumarin-6-labeled T7-P-LPs distributed much more extensively in the region of the brain than that of P-LPs (Fig. 7A). This indicated that unlike P-LPs, the T7-P-LPs penetrated into the brain parenchyma through the endothelial cells of BBB, and proved that the most likely transport mechanism was transferrin receptor mediated transcytosis process. The enhanced accumulation of T7-P-LPs in brain encouraged us to use it as a targeting drug delivery system for ZL006 to treat ischemic stroke.

### *Ex vivo* fluorescent image of ischemic brain

To validate the ability of active targeting to ischemic stroke animal model brain, we performed *ex vivo* imaging of the ischemic brains. DiR-labeled P-LPs and T7-P-LPs were injected into the MCAO rats immediately after the operation. The fluorescence intensity of different DiR-labeled liposomes in ischemic brain slices is shown in Fig. 7B. The qualitative results showed that a higher uptake efficacy of DiR-labeled T7-P-LPs than that of P-LPs in ischemic brain sections at 6 h or 24 h, which provided the evidence that T7-modified liposomes could effectively and consistently achieve the active targeting to the ischemic brain *via* transferrin receptor mediated transcytosis.

### Biodistribution of liposomes in mice

**Figure 8:**
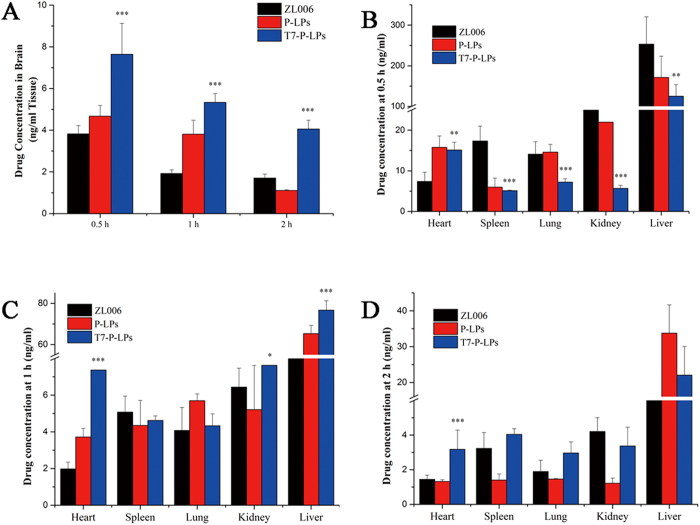
Brain (**A**) and other tissue biodistribution (**B–D**) of ZL006, P-LPs/ZL006 and T7-P-LPs/ZL006 after intravenous inject to ICR mice at 0.5, 1 and 2 hours. Data are represented with mean ± SD (n = 4). *p < 0.05, **p < 0.01, ***p < 0.001 compared with ZL006 and P-LPs/ZL006. P-LPs: P-LPs/ZL006; T7-P-LPs: T7-P-LPs/ZL006.

The distribution into various organs of free ZL006, P-LPs/ZL006 and T7-P-LPs/ZL006 at 0.5, 1 and 2 h after intravenous administration are shown in Fig. 8. At 0.5, 1 and 2 h, the drug concentration of T7-P-LPs/ZL006 group was 2.00, 2.77 and 2.38 fold over that of free ZL006 group in the brain tissue, respectively (Fig. 8A). Compared with P-LPs/ZL006 and free ZL006, T7-P-LPs/ZL006 exhibited a significant increase of drug accumulation in the brain tissue due to its better brain targeting delivery.

In addition, the biodistribution of the three formulations into other major organs (heart, spleen, kidney, lung and liver) is shown in Fig. 8B–D. The drug distribution was widely and rapidly. The maximum concentration appeared in liver at 0.5 h after administration, and followed the order: liver >kidney >heart> lung> spleen. It was noteworthy that a large amount of free ZL006 (253.13 ± 37.62 and 28.96 ± 1.40 ng/mL) accumulated in the liver and kidney, respectively. However, the concentration of P-LPs/ZL006 and T7-P-LPs/ZL006 in liver was 151.25 ± 52.36 and 124.92 ± 28.58 ng/mL, and the concentration of two liposomes in kidney was 18.99 ± 6.38 and 15.68 ± 0.75 ng/mL, respectively. Compared with free ZL006, P-LPs/ZL006 and T7-P-LPs/ZL006 exhibited a significant decrease of drug accumulation in the liver and kidney. It is common knowledge that liver and kidney are main organs of drug metabolism. According to the accumulation of ZL006 in liver and kidney, it provided the evidence that P-LPs/ZL006 and T7-P-LPs/ZL006 could reduce drug metabolism.

### Effect of liposomes on infarct volume and neurological deficiencies

**Figure 9:**
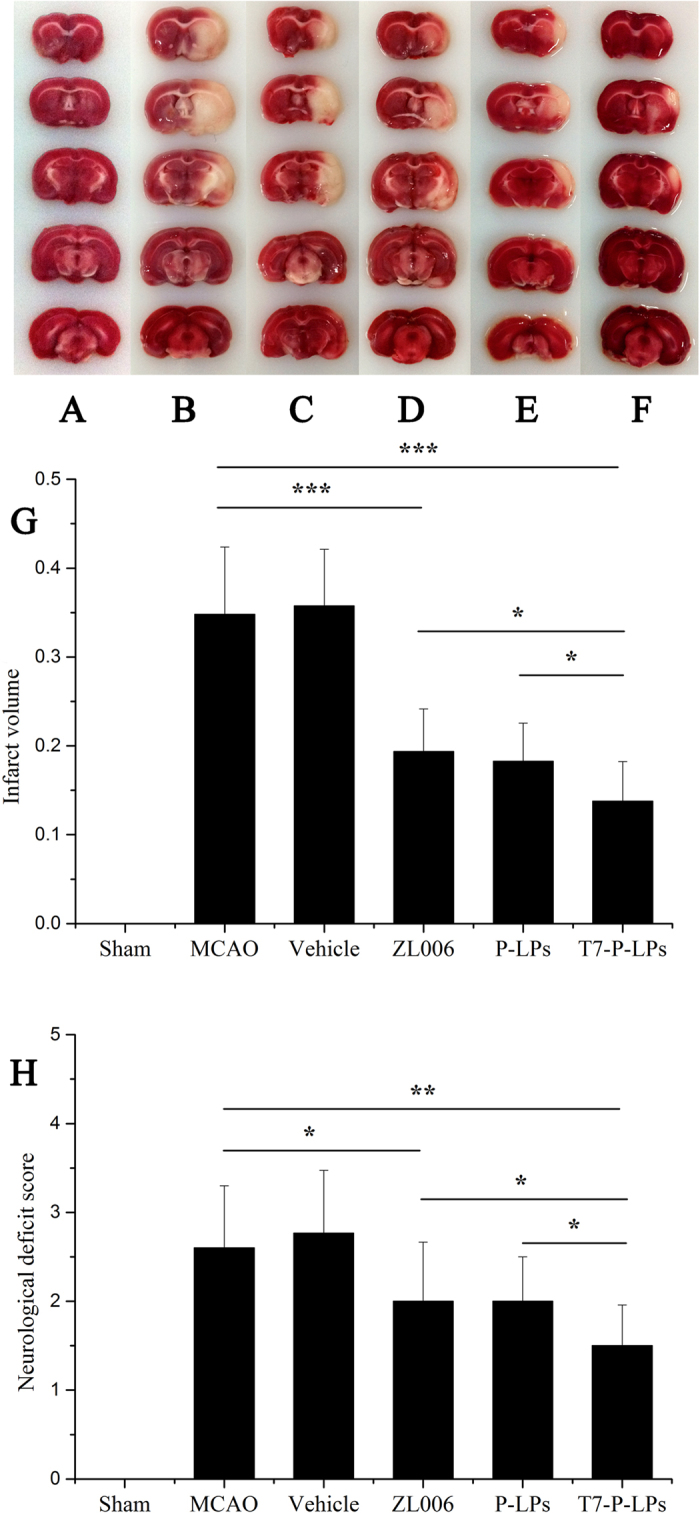
Effect of T7-P-LPs on brain infarct volume and neurological deficits at 24 h after MCAO in rats. Representative TTC-stained brain sections of Sham-operated group (**A**), MCAO group (**B**), Vehicle group (**C**), free ZL006 group (**D**), P-LPs/ZL006 group (**E**) and T7-P-LPs/ZL006 group (**F**) were shown in the figure. The non-ischemic region is red, and the infarct region appears in white. And quantification of brain infarct volume (**G**), Neurological scores of rats after cerebral ischemia (**H**) were shown. Data are expressed with mean ± SD (n = 9). *p < 0.05, **p < 0.01, ***p < 0.001 compared with MCAO group, Vehicle group and ZL006 group. Vehicle: vehicles-T7-P-LPs; ZL006: free ZL006; P-LPs: P-LPs/ZL006; T7-P-LPs: T7-P-LPs/ZL006.

The infarct volume and neurological deficit scores in response to ischemic injury were examined 24 h after MCAO. Rats in the sham-operated group had no infarct and neurological deficit score (Fig. 9A,G,H). By contrast, the brain infarct volume and neurological deficit score in MCAO group were significantly higher (Fig. 9B,G,H). As observed in Fig. 9F,G,H, treatment with T7-P-LPs/ZL006 obviously decreased infarct volume and ameliorated neurological deficit induced by ischemia reperfusion compared with that of free ZL006 group (P < 0.05, Fig. 9D,G,H). However, no significant difference in infarct volume and neurological deficit was found between the MCAO group and vehicle group (Fig. 9B,C,G,H). These results suggested that vehicle group did not display protective effect. Furthermore, T7-P-LPs/ZL006 displayed more protective effect than equivalent dose of free ZL006 on the surgical MCAO injury.

### *In vivo* preliminary safety evaluation

**Figure 10:**
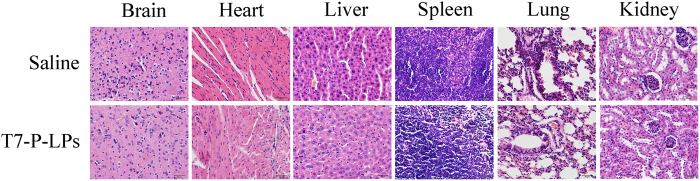
Histochemistry analysis of brain, heart, liver, spleen, lung and kidney section stained with hematoxylin eosin of ICR mice 24 h after i.v. administration of 100 mg/kg saline and T7-P-LPs for 7days, one dose per day. Bar: 20 μm.

The toxicity of liposomes for application in biomedicine is always an important concern. For safety purpose, we evaluated systematic toxicity of T7-P-LPs in healthy ICR mice after intravenous injection of T7-P-LPs at a dosage of 100 mg/kg one dose per day for seven days. Compared with the saline group, no death and serious body weight loss were found in all test groups during the study period. Major tissues including brain, heart, liver, spleen, lung and kidney have no obvious histopathological abnormalities or lesions in the two groups (Fig. 10). Furthermore, there was no significant difference about the AST, ALT, BUN and creatinine levels between T7-P-LPs and saline group in Table 2. These results indicated that multiple dosing of T7-P-LPs had minimal impact in these tissues, showing that there was no significant inflammatory response caused by T7-P-LPs. Moreover the long-term toxic effects of the T7-P-LPs are required to investigate in the future study.

## Discussion

Liposomes can provide a versatile drug delivery vehicle due to their unique structural and excellent biocompatibility^12^. Previously, researchers have taken advantage of these properties to deliver drugs and gene^13^,^14^. However, plain liposomes are lack of brain active targeting. Therefore, active targeting liposomal delivery system based on receptor mediated endocytosis has achieved great development and provides hope for brain diseases therapy in recent years^15^,^16^. Such receptors target could maximize chemotherapeutic agents into brain and minimize the systemic toxic effects.

In this study, T7 was conjugated to liposome drug delivery system. T7-P-LPs was designed and constructed as a brain-targeted carrier to improve the targeting capability and the therapy of stroke. The results showed that T7-P-LPs exhibited a good brain targeting ability, little cytotoxicity and effective treatment to ischemic stroke.

T7-P-LPs/ZL006 were generally spherical and the particle size was under 100 nm (Fig.2), which could improve the endocytosis by the brain capillary cells and prevent spleen filtering^17^,^18^. Furthermore, T7-conjugated liposomes possessed a favorable brain-targeting quality, which were displayed by cellular uptake and biodistribution experimentation *in vitro* and *in vivo*, respectively. The cellular uptake characteristic of liposomes was investigated both qualitatively and quantitatively. The results demonstrated an active targeting role of peptide T7 in coumarin-6-labeled T7-P-LPs uptake by BCECs (Figs 3, 4). As showed in Fig. 7, the drug concentration of T7-P-LPs/ZL006 group was significantly higher than that of free ZL006 group in the brain tissue. The targeting mechanism of T7-P-LPs/ZL006 to BBB may be based on T7 specific binding to TfR which is over-expressed on the brain capillaries endothelial cells. Moreover, PEGylation rendered liposomes longer circulatory time in plasma and better biocompatibility^19^,^20^.

It is well known that BBB is a dynamic barrier protecting the brain against invading organisms. Furthermore, although there is a compromised endothelial barrier which facilitates molecular transport under some disease conditions such as ischemic stroke, BBB is still present in the infiltrating margin of brain diseases. Therefore, we developed T7 peptide modified ZL006 loading liposome to improve the drug delivery to brain and enhance the therapeutic effect against ischemic stroke for ZL006. Firstly, we performed imaging of the healthy rats to evaluate the ability of T7-P-LPs across BBB *in vivo*. Then, *ex vivo* imaging of the ischemic rats was to evaluate the ability of T7-P-LPs across BBB after brain damage. These results showed that T7-modified liposomes could effectively and consistently achieve the active targeting to the both intact and ischemic brain, suggesting that T7 peptide obviously increased the efficiency of uptake of liposomes by the brain before and after stroke (Fig. 6A,B). In addition, the pharmacodynamic study further demonstrated that T7-P-LPs/ZL006 significantly reduced infarct volume and ameliorated neurological deficit induced by ischemia reperfusion compared with free ZL006 (Fig. 8). Clearly, targeting to the brain is still far from pretty, further work is needed to accurately deliver drugs to the site of cerebral infarction.

## Methods

### Materials

ZL006 was kindly provided by Prof. F Li, Department of Medicinal Chemistry, School of Pharmacy, Nanjing Medical University, Nanjing, China. Modified 7-amino acid peptide (Cys-HAIYPRH, named as T7) was purchased from Shanghai GL Biochem Ltd (Shanghai, China). Mal-PEG-DSPE was purchased from Laysan Bio Co., USA. BCA kit. TritonX-100 and MTT were purchased from Beyotime Biotechnology Co., Ltd. (Nantong, China). The other chemical reagents were of analytical grade and used as received.

### Animals and cell line

Spraguee-Dawley (SD) rats (male, 5–6weeks, 200 ± 20 g) and ICR mice (male, 4–5weeks, 20 ± 2 g) were supplied by Animal Experimental Center of Nanjing Medical University (Nanjing, China). All animal experiments were carried out in accordance with guidelines evaluated and approved by the ethics committee of Nanjing Medical University (Nanjing, China).

BCECs, the brain capillary endothelial cells from rats were cultured in DMEM medium supplemented with 20% FBS at 37 °C in a 5% CO_2_ humidified atmosphere.

### HPLC analysis

The concentration of ZL006 in samples was measured via HPLC conducted by using a LC-10ATVP pump and SPD-10AVP UV detector (Shimadzu, Kyoto, Japan). The mobile phase was consisted of methanol and ammonium acetate buffer solution (0.25 mol/L, pH 6.0) (35:65, v/v) and the flow rate was set at 1.0 mL/min and the detection wavelength was 284 nm. The HPLC was calibrated with standard solutions of 1–100 μg/mL of ZL006 dissolved in methanol (Y = 48030X-17645, correlation coefficient of R^2^ = 0.9997). The limit of quantification was 0.5 ng/mL, and the coefficients of variation were all within 3.5%.

### Synthesis and characterization of T7-PEG-DSPE

T7 was conjugated to the PEG-DSPE by the reaction of maleimide with thiol group (-SH) as previously described with minor revision^21^,^22^. Firstly, Male-PEG-DSPE was dissolved in 1 mL dimethylformamide (DMF), and then added into 10 mL phosphate buffer solution (pH 7.4). Secondly, the linear T7 peptide was incubated with the formed solution for 6 h at room temperature. Finally, the resulting T7-PEG-DSPE was purified by dialysis (MWCO 3.5 kDa) against distilled water and obtained by freeze drying. ^1^H NMR and FTIR of T7-PEG-DSPE were characterized.

### Preparation of T7-P-LPs

Liposomes loaded with ZL006, coumarin-6-labeled liposomes and DiR-labeled liposomes were prepared respectively for different uses by the ethanol injection method^23^,^24^. For T7-P-LPs/ZL006, the mixture of soya lecithin/cholesterol/PEG-DSPE/T7-PEG-DSPE/ZL006 (40:10:9:2:8, w/w) were dissolved in ethanol, and then added dropwise into the constant stirring external buffer phase (pH 4.0) using a syringe at 50 °C. After that, the particle size of the crude dispersion was minimized by intermittent probe sonication (Scientz-IID, Shanghai, China). Ethanol was then evaporated with a vacuum rotary evaporator. Finally, the liposomal suspension was filtered with 0.45 μm and 0.22 μm filters, respectively. All the procedures were conducted in darkness. Coumarin-6-labeled liposomes and DiR-labeled liposomes were prepared using the same method.

### Morphology, particle size and zeta potential

The morphology of T7-P-LPs/ZL006 was investigated using a transmission electron microscope (TEM) (JEOL USA, Wilmington, DE, USA). The particle size and zeta potential of the liposomes were determined by the Malvern Zetasizer Nano ZS90 instrument (Malvern, UK).

### Encapsulation efficiency and loading capacity of T7-P-LPs/ZL006

It had been found that the solubility of ZL006 in acetate buffer (CAS, pH 4.0) was less than 1 μg/mL in our preliminary experiment. So, the amount of free drug which was filtered by 0.45 μm and 0.22 μm in T7-P-LPs/ZL006 could be ignored. The encapsulation efficiency (EE) and loading capacity (LC) of T7-P-LPs/ZL006 were calculated according to the flowing equations:


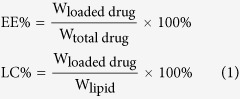


where W_loaded drug_, W_total drug_ and W_lipid_ are the amount of ZL006 in the liposomes after filtered with 0.22 μm filter, the total amount of ZL006 in the liposomes after evaporated and the mass of soya lecithin and cholesterol, respectively.

### Cellular uptake by BCEC cells

#### 1) Fluorescent microscopy of coumarin-6 labeled liposomes uptake by BCECs

Qualitative analysis of cellular uptake of T7-P-LPs was performed via fluorescent microscopy. The BCECs were incubated with coumarin-6-labeled liposomes for 1 h at different concentrations. After washing with cold PBS (pH 7.4), the cells were visualized using a fluorescent microscope (Imager A1, Zeiss, Germany).

#### 2) Quantitative analysis of coumarin-6 labeled liposomes uptake by BCECs

For quantitative experiment, the cells were incubated with diffident liposomes in culture medium for 1 h at different concentrations. Furthermore, in order to study the effects of incubation time on cellar uptake, BCECs were incubated with 100 μg/mL liposomes for 0.25, 0.5, 1, 1.5, 2, 3, 4, and 6 h at 37 °C, respectively. At the end of incubation, samples were removed and washed with cold PBS. The cells were lysed by 400 mL of 1% TritonX-100 per well for 10 min. An aliquot of the cell lysate from each well was used to investigate the total cells protein content using the BCA protein assay^25^. The fluorescence intensity of the cell lysates was measured by HPLC with fluorescence detector for analysis of coumarin-6 content.

#### 3) Quantitative analysis of ZL006 loaded liposomes uptake by BCECs

BCECs were seeded at a density of 1 × 10^5^ cells/well in 24-well plates and incubated for 24 h. After washed with PBS, BCECs were incubated with ZL006 loaded T7-P-LPs, P-LPs and free ZL006 at different concentration for 30 min at 37 °C. For the competition assay, free peptide-t7 was added to the wells in advanced at a concentration of 500 μg/mL. After 30 min pre-incubation at 37 °C, the compounds were withdrawn from the wells, and 200 μg/mL of T7-P-LPs/ZL006, P-LPs/ZL006 and free ZL006 were added and incubated for 30 min, respectively. In a separate experiment, to study the saturated concentration of ZL006 loaded liposomes, the medium was replaced with a series of concentration (100–600 μg/mL) of T7-P-LPs/ZL006, P-LPs/ZL006 and free ZL006, respectively. The plate was incubated for 30 min at 37 °C.

At the end of incubation, samples were removed and washed with cold PBS. The cells were lysed by 400 mL of 1% TritonX-100 per well for 10 min. An aliquot of the cell lysate from each well was used to investigate the total cells protein content using the BCA protein assay. The ZL006 intensity of the cell lysates was measured by HPLC with UV detector for analysis of ZL006 content.

### MTT cell viability assay

The *in vitro* toxicity of liposomes with or without ZL006 was evaluated on BCECs by MTT assay as described previously^26^,^27^. BCECs were seeded in 96-well plates in 200 μL of DMEM medium to obtain a concentration of 2000 cells per well, and incubated for 24 h. The medium in each well was then incubated for 72 h with 200 μL medium containing blank vehicle, P-LPs/ZL006, T7-P-LPs/ZL006 and ZL006 (free drug dissolved in DMSO) with a series of concentrations ranging from 0.001 to 100 μg/mL. The MTT absorbance at 570 nm of each well was measured by a microplate reader (ELX800, Biotek, USA).

### *In vivo* penetration across BBB of T7-P-LPs

To determine the efficiency of *in vivo* penetration across BBB into the brain, coumarin-6-labeled liposomes were infused through the tail vein of rats. 2 h after administration, the rats were sacrificed and the brains were collected after perfusion with normal saline and paraformaldehyde respectively. After anhydration, the brains were cut into 5 μm slices by frozen section. Then, the brain slices were examined under a fluorescent microscope (Imager A1, Zeiss, Germany) using a fluorescein isothiocyanate filter (excitation/emission, 495/520 nm)^28^.

### *Ex vivo* fluorescent image of ischemic brain

To determine the efficiency of uptake of different liposomes into the ischemic brain, DiR-labeled liposomes were infused through the tail vein of rats which were subjected to MCAO model. The MCAO rat model was established as previously^29^. Male SD rats were anesthetized with 10% chloralic hydras and the common carotid artery (CCA), the internal carotid artery (ICA) and the external carotid artery (ECA) were isolated after a median incision of the median neck skin. After that, a MCAO monofilament was inserted into the ECA through the ICA then advanced to the middle cerebral artery (MCA), causing a blockage of blood flow in the MCA. After 2 h, reperfusion was initiated by removing the monofilament. Then, 6 or 24 h after MCAO, the brain slices were obtained. After washed with saline, the brain sections were imaged using bioluminescence imaging system (IVIS Spectrum System, Caliper Life Sciences, USA) equipped with filter sets (excitation/emission, 748/780 nm) for fluorescence^30^.

### Biodistribution of ZL006 loading liposomes in mice

ICR mice weighting 20 ± 2 g were divided into three groups at random (n = 12). Free ZL006, P-LPs/ZL006 and T7-P-LPs/ZL006 (all containing ZL006 4 mg/kg) were administered to each group through intravenous route, respectively. At designated time intervals (0.5, 1 and 2 h), the mice were executed and the major organs samples including brain, heart, liver, spleen, lung and kidney were collected. Before pretreatment, these tissues were rinsed with cold saline solution to remove the blood and then blotted with paper towel. Protein precipitation of the samples was performed before analysis^31^. Then the samples were injected into the LC–MS/MS systems for analysis. The LC–MS/MS system consists of an Agilent Series 1200 HPLC system (Agilent Technologies, Waldbronn, Germany) and a 6410 Triple Quad LC/MS mass spectrometer (Agilent Technologies, Santa Clara, CA, USA). The data was collected and processed using the Agilent MassHunter Workstation Software (version B.01.04).

### Anti- ischemic stroke

In this study, rats were randomly divide into six groups (n = 10): group 1: sham-operated group injected with normal saline in a comparable volume; group 2: model group; group 3: vehicle control group administered with blank T7-P-LPs; group 4: treated with free ZL006 (4 mg/kg); group 5: treated with P-LPs/ZL006 (4 mg/kg); group 6: treated with T7-P-LPs/ZL006 (4 mg/kg). All formulations were administered *via* the intravenous route at the time of reperfusion following 2 h of ischemia, respectively.

Neurological behavior was evaluated at 24 h after MCAO. The neurological deficit score was assessed using an established five point scale^32^. After the neurological assessment, rats were sacrificed and brains were harvested. The brains were chilled in ice-cold saline for 5 min, and then sectioned into 2 mm-thick coronal slices which were stained with 2% 2,3,5-triphenyltetrazolium chloride (TTC) at 37 °C for 30 min and then immersed in 4% paraformaldehyde (PFA) solution for preservation. TTC-stained sections were photographed and the digital images were analyzed using image tool 3.0, then the infarct area percentage was calculated using the following equation:


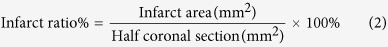


### *In vivo* preliminary safety evaluation

Ten male ICR mice were randomly divided into two groups (n = 5). Each group received an intravenous injection of blank T7-P-LPs (100 mg/kg) or saline at one dose per day for seven days. The body weight was measured each day. Blood sample and brain tissue were collected at 24 h after the last administration for hematologic and histochemistry analysis. The serum aspartate transaminase (AST), alanine transaminase (ALT), urea nitrogen (BUN) and creatinine levels were assayed using Hitachi 7080 Chemistry Analyzer (Hitachi Ltd., Japan). The brain, heart, liver, spleen, lung and kidney tissue were fixed with paraformaldehyde for 48 h and embedded in paraffin. Each section was cut into 5 mm, processed for routine hematoxylin and eosin (H&E) staining, and then visualized under fluorescent microscope (Leica DMI 4000B, Germany).

### Statistical analysis

All the results were expressed as mean ± standard deviation (SD). Statistical analysis was performed with SPSS 20.0 software. Statistical analysis was used one-way ANOVA test. Differences were considered significant when *p < 0.05, **p < 0.01, ***p < 0.001, respectively.

## Additional Information

**How to cite this article**: Wang, Z. *et al.* Enhanced anti-ischemic stroke of ZL006 by T7-conjugated PEGylated liposomes drug delivery system. *Sci. Rep.* **5**, 12651; doi: 10.1038/srep12651 (2015).

## Figures and Tables

**Table 1:** The characterizations of liposomes loaded with ZL006 with or without T7 modification.

Formulation	Particle size (nm)	Polydispersity index (PDI)	Zeta Potential (−mV)	EE%	LC%
T7-P-LPs/ZL006	74.24 ± 2.17	0.154 ± 0.058	16.767 ± 0.874	87.89 ± 3.58	9.76 ± 0.55
P-LPs/ZL006	73.78 ± 2.84	0.167 ± 0.054	15.400 ± 2.066	85.37 ± 2.06	9.40 ± 0.45

Data are represented with mean ± SD (n = 3).

**Table 2:** Mice serum level of biochemical variables after intravenous treatment with T7-P-LPs at concentration of 100 mg/kg for 7 days (n = 4).

Groups	AST	ALT	BUN	Creatinine
	(U/L)	(U/L)	(mmol/L)	(mmol/L)
Saline	15.36 ± 3.07	15.42 ± 4.76	9.20 ± 2.11	68.42 ± 8.82
T7-P-LPs	16.07 ± 3.15	17.55 ± 4.86	9.76 ± 3.76	72.94 ± 15.68
